# Identification of candidate genes and regulatory factors related to growth rate through hypothalamus transcriptome analyses in broiler chickens

**DOI:** 10.1186/s12864-020-06884-5

**Published:** 2020-07-23

**Authors:** Katarzyna Piórkowska, Kacper Żukowski, Katarzyna Połtowicz, Joanna Nowak, Katarzyna Ropka-Molik, Natalia Derebecka, Joanna Wesoły, Dorota Wojtysiak

**Affiliations:** 1 Department of Animal Molecular Biology, National Research Institute of Animal Production, Balice, Poland; 2 Department of Cattle Breeding, National Research Institute of Animal Production, Balice, Poland; 3 Department of Poultry Breeding, National Research Institute of Animal Production, Balice, Poland; 4 Adam Mickiewicz University, Faculty of Biology, Laboratory of High Throughput Technologies Institute of Molecular Biology and Biotechnology, Poznań, Poland; 5 Department of Animal Genetics, Breeding and Ethology, University of Agriculture in Krakow, Cracow, Poland

**Keywords:** Growth rate, Broilers, RNA-seq, Hypothalamus response

## Abstract

**Background:**

Intensive selection for growth rate (GR) in broiler chickens carries negative after-effects, such as aberrations in skeletal development and the immune system, heart failure, and deterioration of meat quality. In Poland, fast-growing chicken populations are highly non-uniform in term of growth rate, which is highly unprofitable for poultry producers. Therefore, the identification of genetic markers for boiler GR that could support the selection process is needed. The hypothalamus is strongly associated with growth regulation by inducing important pituitary hormones. Therefore, the present study used this tissue to pinpoint genes involved in chicken growth control.

**Results:**

The experiment included male broilers of Ross 308 strain in two developmental stages, after 3rd and 6th week of age, which were maintained in the same housing and feeding conditions. The obtained results show for the overexpression of genes related to orexigenic molecules, such as neuropeptide Y (*NPY*), aldehyde dehydrogenase 1 family, member A1 (*ALDH1A1*), galanin (*GAL*), and pro-melanin concentrating hormone (*PMCH*) in low GR cockerels.

**Conclusion:**

The results reveal strong associations between satiety centre and the growth process. The present study delivers new insights into hypothalamic regulation in broiler chickens and narrows the area for the searching of genetic markers for GR.

**Graphical abstract:**

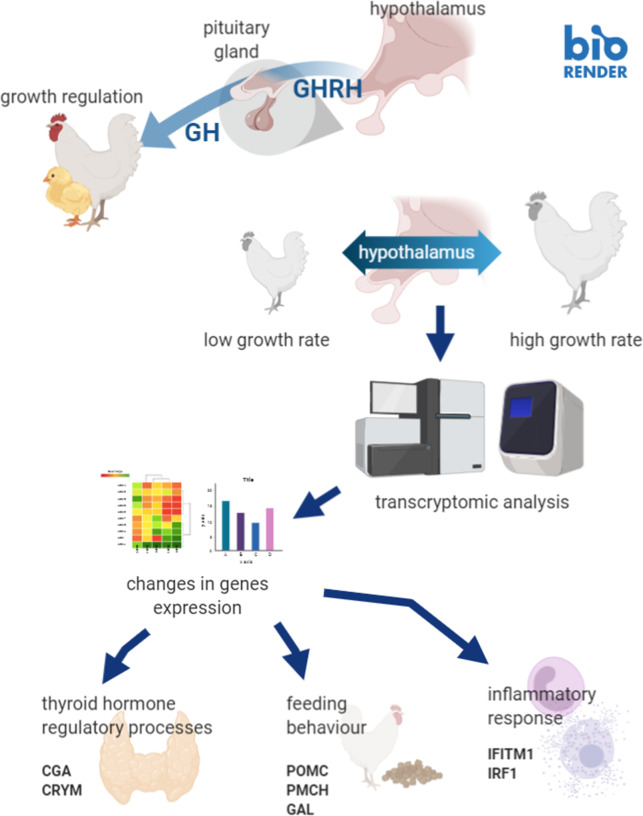

## Background

The broiler chicken industry has grown over its 60-year history. The Global Information and Early Warning System (GIEWS) reported that the world poultry meat output was estimated at 121.6 million tons in 2018 with an upward tendency [1]. The increasing demand for poultry was an engine to bred highly efficient chicken populations with significantly improved productive values [2]. The conducted selection aimed to improve the growth rate, feed efficiency and carcass meat content [3]. The market broiler mass after sixth weeks from hatching has increased 4-fold, and pectoralis muscle nearly 2-fold over 25 years [4]. Unfortunately, the rate of genetic changes resulted in the alternations in bird physiology [5]. In turn, intensive selection led to negative after-effects, such as aberrations in skeletal development [6] and the immune system [7], heart failure [8] and deterioration of meat quality [9] what reflects elevated attendance of meat defects in fast-growing populations. The most frequent poultry meat defects are deep pectoralis myopathies (DPM) [10]; white striping; Pale, Soft and Exudative (PSE) meat [11], wooden breast [12]; and intramuscular connective tissue, termed spaghetti meat [13].

Ross 308 is the world’s most-popular commercial broiler hybrid. Ross 308 chickens are characterised by rapid growth and high muscle mass developed at an early age, providing the possibility of early slaughtering. Usually, they are slaughtered at sixth weeks from hatching, which is associated with the attainment of an optimum balance between growth rate, feed efficiency, high muscle mass, high performance and feather maturity. Regardless of the flock age, most of Ross 308 populations show low uniformity of growth rate (GR), where GR is estimated as average gain in body weight (BW) for a defined period of rearing. The differences are already visible in the first weeks of life, and after six-weeks, they can even reach 1.5 kg in the BW in one broiler population, which is unprofitable for poultry producers, butchers, and the future processing. A similar observation was also described in different broiler strains [14, 15].

On the other hand, the hypothalamus plays an overarching role in the regulation of primary metabolic processes also in bird organisms. It controls body temperature, hunger, sleep, growth and circadian rhythms, by stimulating the pituitary which secretes essential hormones controlling other glands/organs. As an example, the hypothalamus releases somatoliberin (GHRH) that induces pituitary somatotropin (GH) secretion - most crucial growth factor. Therefore, the hypothalamic molecular activity is regulated by numerous exogenous stimuli [16]. Thus, the newest high-throughput molecular genetic tools are used for the determination of these processes. Kamineni [17] in his doctoral dissertation used RNA-sequencing based on next-generation sequencing (NGS) technology to show that heat stress in broiler chickens increases hypothalamic gene expression of rate-limiting enzymes, such as sterol regulatory element-binding transcription factor 1 (SREBF1) and lipoprotein lipase (LPL). In turn, Han et al. [18] using analysis of miRNAom suggested that miRNAs play an essential role in the hypothalamus for timing the rapid development of chicken gonads. While Piórkowska et al. [19] used the RNA-seq method to pinpoint the hypothalamic gene expression changes during postnatal development in broiler chickens.

The present study aimed to identify whether different growth rate in broiler chickens found the reflection in hypothalamic genes’ expression and the activation of the essential molecular processes. This approach enables to narrow the search area of potential genetic markers associated with the determination of growth in broilers. The present experiment used transcriptome analysis based on next-generation sequencing technology.

## Results

### Cockerel characteristics

During the experiment, fattening and slaughter traits for all chickens were recorded. The high growth rate (HGR) cockerels were 176 g and 685 g heavier than low growth rate (LGR) birds, after the 3rd and 6th week from hatching, respectively (Table 1). The growth rate did not affect fat level but influenced leg and breast muscle mass. Moreover, low growth rate (LGR) cockerels slaughtered in 3rd and 6th week of age took 55 g and 461.2 g less feed respectively and showed a higher feed conversion ratios (FCR), which were measured from 2nd week of age to the slaughter.

**Table 1:** Traits of investigated cockerels used in RNA sequencing and qPCR analyses

**Trait**	**ROSS308**			
	**3-week-old**		**6-week-old**	
	**LGR** (*n* = 9)	**HGR** (*n* = 9)	**LGR** (*n* = 9)	**HGR** (*n* = 9)
	Mean ± SD	Mean ± SD	Mean ± SD	Mean ± SD
BW in first day (g)^a^	45.1 ± 1.72	45.4 ± 1.64	44.9 ± 1.14	45.2 ± 2.04
BW in 2nd week (g)	486.67 ± 10.0^A^	547.0 ± 14.94^B^	484.0 ± 11.74^A^	545.6 ± 11.30^B^
Slaughter BW (g)	1120.0 ± 14.14^A^	1296.0 ± 20.66^B^	3222.0 ± 75.10^A^	3906.7 ± 95.92^B^
Average daily gain (g)^a^	51.19 ± 0.69^A^	59.55 ± 0.92^B^	72.21 ± 1.71^A^	87.76 ± 2.18^B^
Average daily gain from 2nd week (g)	79.17 ± 2.34^A^	93.17 ± 2.39^B^	88.32 ± 2.58^A^	108.42 ± 3.23^B^
Breast muscles (g)	200.18 ± 11.27^A^	243.74 ± 11.94^B^	786.45 ± 66.92^A^	955.40 ± 46.44^B^
Leg muscles (g)	174.05 ± 8.97^A^	204.33 ± 11.52^B^	538.98 ± 51.59^A^	657.69 ± 42.71^B^
Abdominal fat (g)	8.42 ± 2.63	9.77 ± 2.34	26.59 ± 10.21	34.11 ± 6.33
Feed intake (g)	634.8 ± 18.64^A^	689.8 ± 15.75^B^	4575.6 ± 187.24^A^	5036.8 ± 106.28^B^
Feed Conversion Ratio (FCR)	1.0 ± 0.03^A^	0.92 ± 0.02^B^	1.67 ± 0.07^A^	1.5 ± 0.03^B^

A, B Values in rows with different letters show significant results (*P* ≤ 0.01). The statistical analysis between high and low growth rate chickens was estimated (using Student’s t-test) separately for 3rd and 6th-week old chickens. *LGR* Low growth rate; *HGR* High growth rate; *BW* Body weight; Feed intake – from 2nd week of age to the slaughter; *FCR* Measured based on feed intake and body weight gain from 2nd week of age to the slaughter; ^a^-from hatching

### Analysis of differentially expressed genes

**Fig. 1:**
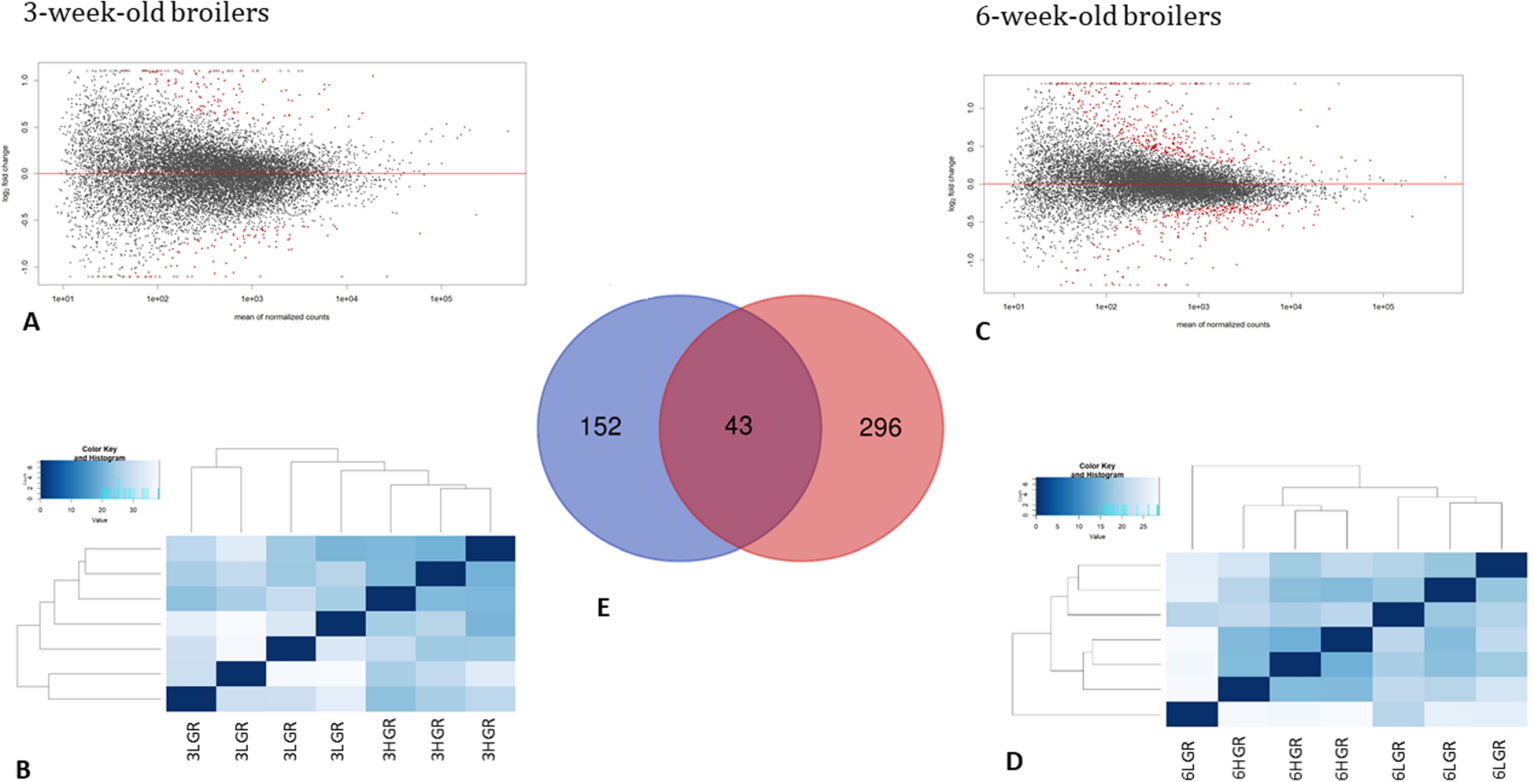
The plots showed hypothalamic DEGs of Ross308 identified in the present analysis. The volcano plots showing the number of significant (red dots) and not-significant (black dots) (**a**) for 3-week-old-chickens and (**c**) 6-week old chickens. The heatmap Principal Component Analysis (PCA) showing sample clustering based on normalised reads for Ross308 broiler chickens characterised high (HGR) and low growth rate (LGR) (**b**) for 3-week old chickens and (**d**) for 6-week-old chickens. On heatmap were indicated seven samples per age groups, because two of them were pinpointed during clustering analysis that are distinct from particular groups, similarly as 6LGR sample that was also removed from further processing. Venn diagram presented DEGs (**e**) identified for 3-week-old, and 6-week-old chickens and 43 shared DEGs

Over 90% of the processed reads were mapped to the chicken reference genome (GCA_000002315.5) included 70% to the exonic regions and 9% to the introns (see supplementary File S1). The clustering analysis excluded three samples from further processing, one of 3-week-old and two of the 6-week-old group (Fig. 1 b, d). The statistic clustering analysis showed that these samples were significantly distinct than the rest samples in the particular groups, which could be a consequence of unknown factor. The GR-dependent DEG analysis was performed using the DESeq2 method, which pinpointed for 43 shared DEGs between both age groups (Fig. 1 a, c, e). They encode proteins involved in luteinizing and follicle-stimulating hormone secretion (*TBX3* and *CGA*), feeding behaviour (*OXT, NPY, PMCH*, and *BSX*), and defence response to the virus (*OASL, MX1, IFITM3, IFIT5*, and *IFI6*).

The hypothalamic response related to changes in growth rate indicates 195 and 339 DEGs (fold-change ≥1.5, by adjusted *P*-value ≤0.05) in 3- and 6-week-old cockerels, respectively (goo.gl/4XX5mt). In younger LGR cockerels, increased expression of genes encoding proteins mainly involved in the organ development (*BSX, MGP, MMP2, MYH11, EGR1, PCDH15, FST, LECT2, TBX3,* and *NPPC*), thyroid hormone regulation process (*CRYM, CGA,* and *CPQ*), regulation of hormone levels (*POMC, SLCO1C1, EGR1, GHR, CGA, TBX3, BLK, GAL, SLC30A8, BTK, ALDH1A1, DIO2, TTR,* and *NR5A1*) and feeding behaviour (BSX, PMCH, NPW, NMU, and NKX2–1) (Table 2) were observed. In turn, DEGs of older LGR birds are involved in the activity of inflammatory response processes included a response to interferon-gamma (*IRF9, CCL19, IFITM1, GBP1, IRF8, IRF7,* and *IRF1*), T and B cell activation (*RAC2, DMB2, CD3E, B2M, CD3D, LCK, CD3E, PTPRC, FOS, BLNK, BTK*, and *PTPN6*), and antigen presentation: folding, assembly and peptide loading of class I (*ERAP1, BFIV21, B2M, BF1,* and *TAP2*), as well as, different signalling pathways regulating hormone levels (*BLK, TTR, CGA, POMC, SLCO1C1, DIO2, EGR1, ALDH1A1, SLC30A8, NR5A1, TBX3, GHR,* and *GAL*) and feeding behaviour (*GAL, FOS, PMCH, BSX, OXT*, and *STRA6*). Three genes showing the highest FC were *ALB, MGAM, FGB* and *AVD, IFI6, CCL19* in 3- and 6-week-old LGR cockerel (goo.gl/4XX5mt), respectively.

**Table 2:** Functional annotation of hypothalamic DEGs in response to a variable growth rate of cockerels after 3rd and 6th weeks from hatching that was performed based on STRING and PANTHER tools

Gene ontology	***FDR***	No.	3-week-old	6-week-old
**GO Biological Process COMPLETE**				
GO:0098883 synapse pruning	1.35E-02			*C1QC, C1QB, C1QA*
GO:0045080 positive regulation of chemokine biosynthetic process	2.01E-02			*CD74, MYOD88, EGR1*
GO:0042403 thyroid hormone metabolic process	3.27E-02	3	*CRYM, CGA, CPQ*	
GO:0048513 organ development	0.00216	10	*BSX, MGP, MMP2, MYH11, EGR1, PCDH15, FST, LECT2, TBX3, NPPC*	
GO:0010469 regulation of signaling receptor activity	2.26E-02	7	*GHRH, PMCH, CGA, NMU, POMC, OGN, NPPC*	
GO:0030154 cell differentiation	0.0424	9	*MGP, MMP2, MYH11, EGR1, PCDH15, FST, LECT2, NPPC, TAGLN*	
GO:0009914 hormone transport	2.10E-03 3.88E-03	5 7	*GHRH, CRYM, CGA, SLCO1C1, TBX3*	*SLCO1C1, CGA, TBX3, GAL, SLC30A8, BTK, TTR*
GO:0010817 regulation of hormone levels	2.32E-04 4.95E-02	16 13	*CRYM, TBX3, GHRH, FGB, SLCO1C1, GGA, BCO1, ALDH6, TCF7L2,CGA, CPLX1, EGR1, CPQ, TRH, CHGA, POMC*	*TBX3, NPY, GAL, DIO2, SLC30A8, SLCO1C1, BLK, TCF7L2, CGA, TTR, NR5A1, EGR1, LHCGR, POMC*
GO:0045187 regulation of circadian sleep/wake cycle	0.0193	3		*PER3, KCNA2, GHR*
GO:0002052 positive regulation of neuroblast proliferation	0.0178	3		*OTP, DCT, BF1*
GO:0034341 response to interferon-gamma	6.19E-04	7		*IRF9, CCL19, IFITM1, GBP1, IRF8, IRF7, IRF1*
GO:0060337 type I interferon signaling pathway	2.88E-02	3		*STAT2, STAT1, IRF3*
**GO molecular function complete**				
GO:0071855 neuropeptide receptor binding	5.77E-03	5	*GHRH, NPY, NMU, PMCH, POMC*	
GO:0004029 aldehyde dehydrogenase (NAD) activity	2.43E-02	2	*ALDH1A1, ALDH6*	
GO:001982 oxygen binding	4.77E-02	2	*ALB, HBAD*	
GO:0005179 hormone activity	1.21E-05	11	*OXT1, GHRH, ENSGALG00000043381, RFLB, CNP3, NPY, CGA, TRH, AVP, PMCH, NMU, POMC,*	*OXT1, CNP3, NPY, POMC, CGA, GAL, PMCH, TTR, PNOC, KL, CALCA, TTR, CCK,*
**GO cellular component complete**				
GO:0071855 neuropeptide receptor binding	5.77E-03	5	*GHRH, NPY, NMU, PMCH, POMC*	
GO:0004029 aldehyde dehydrogenase (NAD) activity	2.43E-02	2	*ALDH1A1, ALDH6*	
GO:001982 oxygen binding	4.77E-02	2	*ALB, HBAD*	
GO:0005179 hormone activity	1.21E-05	11	*OXT1, GHRH, ENSGALG00000043381, RFLB, CNP3, NPY, CGA, TRH, AVP, PMCH, NMU, POMC,*	*OXT1, CNP3, NPY, POMC, CGA, GAL, PMCH, TTR, PNOC, KL, CALCA, TTR, CCK,*
**GO cellular component complete**				
GO:0062023 collagen-containing extracellular matrix	1.63E-03	10	*COCH, MMP2, SPARCL1, COL28A1, MGP, COL6A2, COL4A6, COL1A2, COL1A1, EGF*	
GO:0001664 G protein-coupled receptor binding	2.10E-02	13		*PNOC, CCLI10, OXT1, GAL, CCL4, PDYN, CCLI7, NPY, RSPO3, PMCH, POMC, CCL19, ENSGALG00000003309*
**PANTHER GO-Slim Biological Process**				
GO:0050796 regulation of insulin secretion	3.85E-03	3	*CHGA, TCF7L2, TRH*	
GO:0023056 positive regulation of signaling	4.87E-03	6	*GHRH, TCF7L2, TRH*	
GO:0043588 skin development			*DSP, COL1A1, ENSGALG00000029182*	
GO:0001501 skeletal system development	1.35E-03	5	*COCH, COL6A2, COL6A1, COL1A1, ENSGALG00000029182*	
GO:0007631 feeding behavior	0.03E-01	5	*BSX, PMCH, NPW, NMU, NKX2–1,*	*GAL, FOS, PMCH, BSX, STRA6*
GO:0006954 inflammatory response	1.29E-02	8		*CCLI10, CCL4,CCR2, CCLI7, TLR1B, TLR2A, TLR3, CCL19*
**PANTHER Pathway**				
T cell activation	4.02E-04	8		*RAC2, DMB2, CD3E, B2M, CD3D, LCK, FOS, PTPRC, CD74, FOS, SLP76, CD3D*
B cell activation	1.15E-03	7		*BLK, RAC2, BLNK, BTK, PTPN6, FOS, PTPRC*
5-Hydroxytryptamine degredation	8.40E-03	2	*ALDH1A1, ALDH6*	
Integrin signalling pathway	1.32E-02	7	*COL1A2, COL4A6, COL3A1, COL6A1, COL6A2, ACTA2, COL1A1*	
**Reactome pathways**				
R-GGA-1650814.1 Collagen biosynthesis and modifying enzymes	2.25E-03	5	*COL1A2, COL4A6, COL6A1, COL28A1, COL6A2*	
R-GGA-983170.1 Antigen Presentation: Folding, assembly and peptide loading of class I MHC	2.35E-03	5		*ERAP1, BFIV21, B2M, BF1, TAP2*
Integrin cell surface interactions	6.70E-03	6	*FGB, COL6A2, COL4A6, ENSGALG00000026836, ENSGALG00000004946, COL6A1*	
R-GGA-977606.1 Regulation of Complement cascade	6.21E-05	8	*SERPING1, ENSGALG00000030038, C1QC, C1R, CFH, C1S, C1QB, C1QA*	
R-GGA-5686938.1 Regulation of TLR by endogenous ligand	2.58E-02	4	*LY96, BPI, TLR1B, TLR2A*	

Several genes in the hypothalamus of HGR cockerels showed over 3-fold higher expression (*AVP, ARAP1,* and *OXT* for younger and *GBX2, TCF7L2, GRM2*, and *MGAM* for older cockerels).

### Validation of RNA-seq results and qPCR analysis

**Fig. 2:**
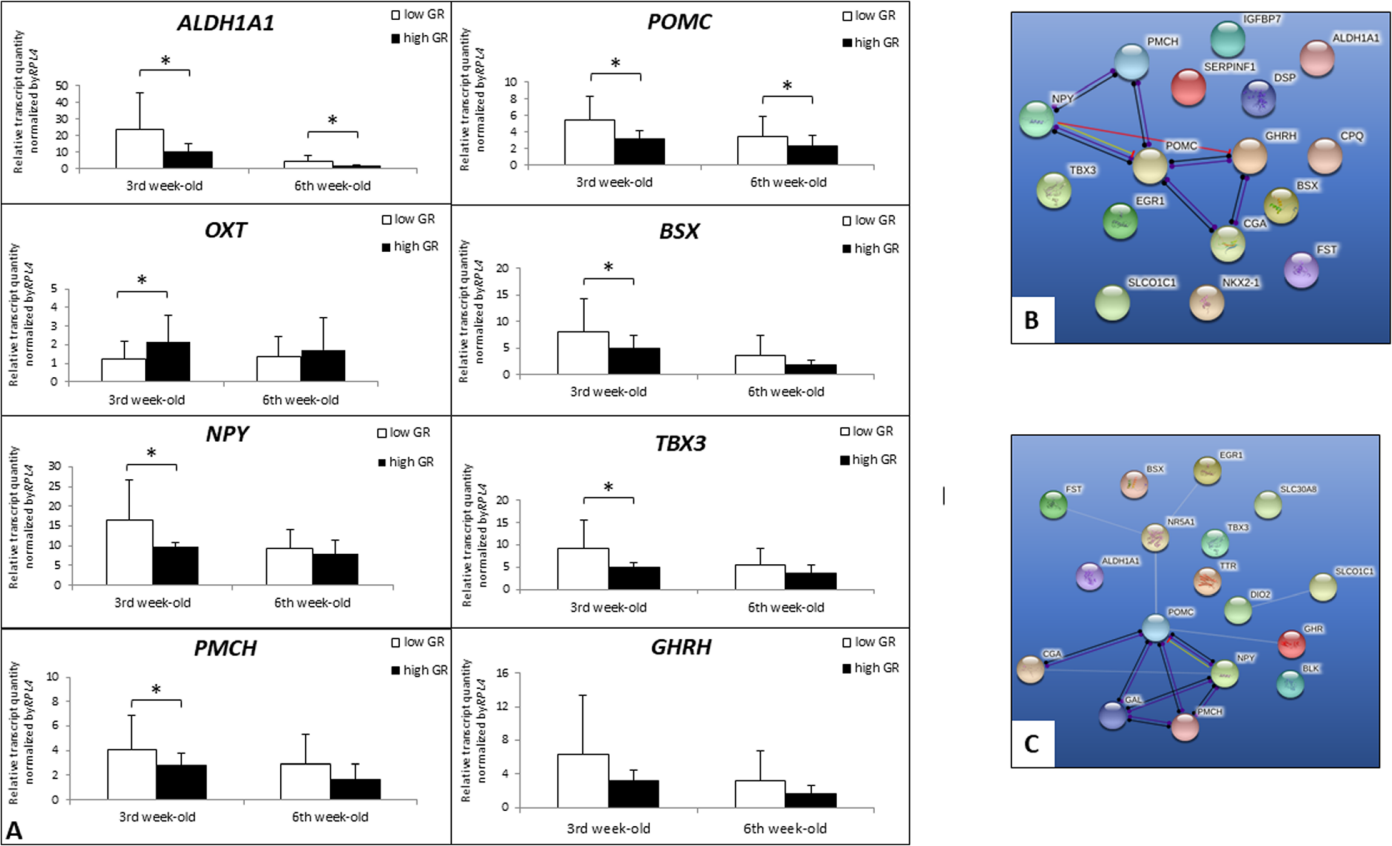
DEGs involved in the regulation of hormonal level. Relative transcript abundance of genes evaluated in the hypothalamus of broilers characterised high (high GR) and low growth rate (low GR) (**a**) and the relationship of genes coding protein involved in the hormonal regulation up-regulated in 3-week-old low growth rate (LGR) cockerels (**b**) and up-regulated in 6-week-old LGR cockerels (**c**). The efficiency of PCR reactions was estimated based on the standard curve method. The gene expression levels were calculated using the delta-delta CT method [20], and the significant differences in gene expression levels between HGR and LGR individuals within each age group were determined by ANOVA (Duncan’s post hoc test; SAS Enterprise v. 7.1 with default settings; SAS Institute, Cary, USA)

Eight DEGs have been validated by the qPCR method and then compared with RNA-seq results using Pearson correlation. The obtained results are accessed by following the link goo.gl/4XX5mt. The lowest R coefficient was 0.82 for *ALDH1A1* (*P*-value = 2.7E-09). Therefore, the qPCR analysis confirmed the RNA-seq results. The comparison between LGR and HGR cockerels groups indicates significantly increased expression of *POMC, ALDH1A1, BSX, PMCH,* and *NPY* genes in LGR birds. In turn, *OXT* gene expression shows the opposite tendency (Fig. 2).

## Discussion

The artificial selection in broiler chickens carried out over the recent decades focused on the increase of bird efficiency. The growth rate was the prime selection trait since the 1950s, and significant progress achieved due to the rise in pectoralis muscle yield and feed efficiency. Currently, broilers reach the slaughter weight faster due to more effective digestion and better energy utilisation during growth [21]. However, the growth rate of broiler chickens is not uniform since the slaughter weight can differ by up to 1.5 kg in one population. In turn, the flock uniformity in ‘live weight’ is a crucial measure of performance when optimising feeding programs of broiler population, as it strongly relates to the yield of processed meat. In many countries, including the USA and Australia, the significant financial losses related to numerous meat defects have been observed [14, 15]. The fast growth in broilers is associated with meat defects that reduce production profits. Therefore in the present study, the idea was born to indicate metabolic and signalling processes that are regulated in the hypothalamus under the influence of variable growth rate in broilers. The goal of the study was to pinpoint the important agents, which may enable control of this economically important chicken trait.

The advantage of the study was that the examined birds were selected from the same chicken population (eggs obtained from one source), maintained under the same feeding and housing conditions to minimalize other than genetic factor. Nonetheless, the differences in BW between the groups were high and constituted 176 and 685 g in the 3rd and 6th week from hatching, respectively. The DEG was performed, and results reveal interesting observation related to neurotransmitters regulation of the feeding depends on ontogenesis stages.

**Fig. 3:**
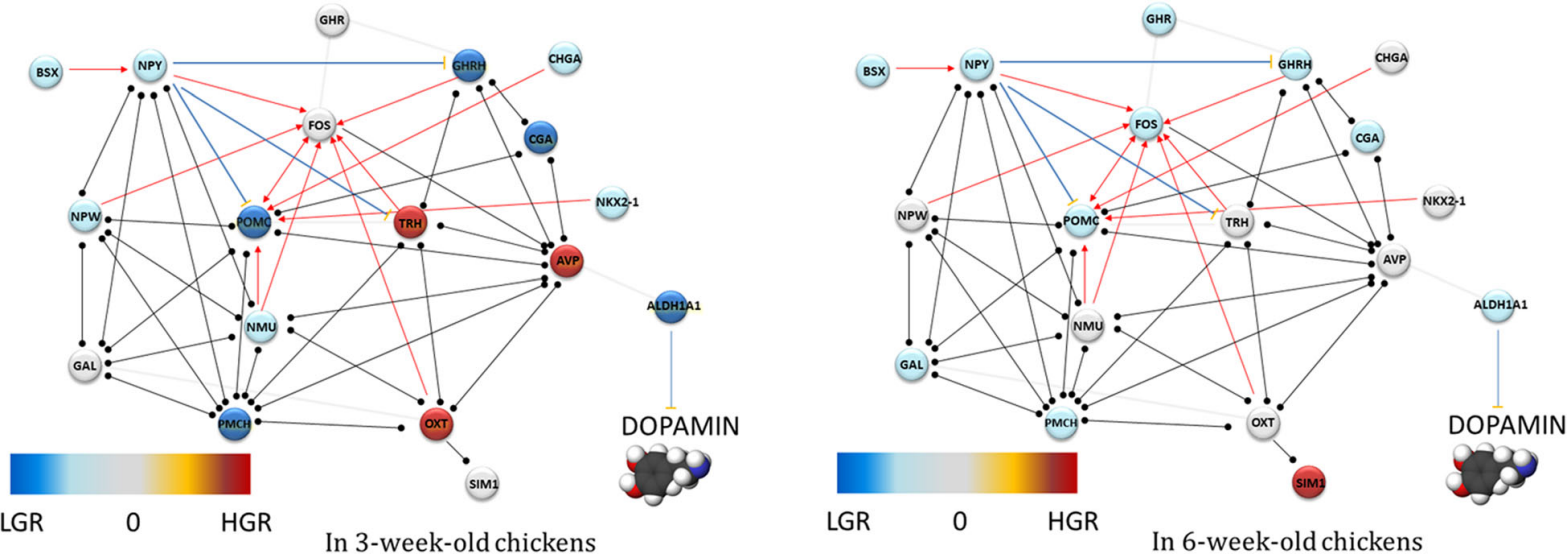
The connection of GR-regulated genes that were associated with feeding behaviour (**a**) in 3-week-old and (**b**) in 6-week-old chickens. The colour saturation is changed along with the level of fold-change, down-regulated genes (orbs) in blue colour, up-regulated in red colour, and in grey colour that were not regulated in response to the growth rate. → inducing, activation; •-• interaction; ⊥ inhibition

*POMC* gene as a precursor for adrenocorticotropic hormones (ACTH), β-endorphin, αmelanocyte-stimulating hormone (αMSH) and βmelanocyte-stimulating hormone (βMSH). It is released by the hypothalamic arcuate nucleus (ARC) and belongs to the anorexigenic molecules [22]. In avians, the regulation by this neurotransmitter is somewhat distinct from that in mammals, since Tachibana et al. [23] observed that broiler chickens artificially selected for their high growth rate were sensitive only to high αMSH doses, which contributed to the suppression of feed intake. In turn, Rice et al. [24] showed an increased *POMC* expression in the hypothalamus of selected high growth rate broilers only after postprandial insulin injection. Honda et al. [25] also suggested that increased feed consumption in broiler chickens could be associated with a lack of β-MSH anorexigenic effect, and an increased level of hypothalamic POMC is not associated with suppressing of food intake. Concerning an ambiguous role of POMC in birds, Boswell and Dunn [26] proposed that avian hypothalamic POMC plays a more significant role in the production of ACTH and β-endorphin than of α-MSH; thus, increased *POMC* expression promotes body mass loss. However, our results show elevated *POMC* mRNA level in the LGR cockerels that characterised by lowered feed intake. This observation supports the thesis of the anorexigenic function of POMC; suppressing hunger and promoting body mass loss. Moreover, in the younger cockerels, positive POMC neuron regulatory element— homeobox protein NK-2 homolog A (Nkx2–1) was found to be overexpressed, and Nkx2–1 ablation from POMC neurons leads to decreased *POMC* expression in adult males and mildly increased their body weight and adiposity [27, 28] (Fig. 3).

Another hypothalamic hormone-neuropeptide Y (NPY) that is associated with the nutritional state was identified as DEG depending on GR. The increased NPY (+ 50%) expression was noticed in the examined LGR cockerels, which was in line with previous studies [29, 30]. The present results show that increased *NPY* expression is associated with lowered feed intake, particularly in younger 3-week-old cockerels. In the same analysed cockerel group, NPY neuron regulatory element— brain-specific homeobox protein (BSX) was found to be overexpressed. The role of BSX was also examined in the loss-of-function study included ob/ob mice [31], where authors confirmed the requirement of BSX for physiological expression of NPY/AgRP and stimuli of hyperphagic response. Therefore the increased BSX expression level could be associated with its NPY regulatory function in avians, as well. Moreover, in 6LGR broiler increased *FOS* expression was observed that encodes c-Fos proto-oncogene. FOS is induced by NPY in insulin companion that action promotes food intake [32]. Further, it is highly probable that TRH mediates the NPY hyperphagia effect via c-FOS [33] (Fig. 3).

ALDH1A1, PMCH, and GAL genes were also differentially expressed in response to different GR and showed increased expression in LGR birds. In the mammal hypothalamus, ALDH1A1 participates in the metabolism of dopamine [34] and decreases its extracellular level. Mebel et al. [35] indicated that mesolimbic dopamine (DA) released in the ventral tegmental area of the brain of mammals implicated in the incentive, reinforcing and motivational aspects of food intake. Moreover, this signalling is sensitive to palatable food with high fat and sugar content that activate DA reward circuitry [36]. However, in chickens, intracerebroventricular injection of dopamine decreases food intake and induces hypophagia [37]. The *PMCH* encodes pre-melanin-concentrating hormone, which is associated with the regulation of feeding behaviour by decreasing energy expenditure and increasing food intake [38]. Sun et al. [39] found in chickens the relationship between a missense *PMCH* variant and shear force measured in the breast and leg muscles, but without effects on growth performance. In the present study, increased *ALDH1A1* and *PMCH* expressions in the LGR chickens at both ages were observed, and between 3rd and 6th week, these differences showed a downward trend. Moreover, the LGR birds showed lowered growth rate and feed intake, what contradicts of the orexigenic function of both molecules in the avian hypothalamus. *GAL* encodes galanin that is believed to be an acute feeding behaviour stimulator in the mammals [40]. Fang et al. [41] described that endogenous galanin contributes to regulating glucose uptake because it decreases insulin resistance and improves its sensitivity. In birds, the role of galanin is not precisely clear since contradictory literature evidence can be found [23]. While, the present study found increased hypothalamic GAL expression in 6LGR broilers, in which low body mass was likely the consequence of low feed intake. The present results deny of orexigenic galanin action at least at this developmental stage in broilers, although Tachibana et al. [40] suggested that galanin function in the vertebrates is constant.

In 3-week-old HGR chickens, increased expression of *AVP* and *OXT* was observed. Both neurotransmitters are known as regulators of nutritional status in the mammals [42, 43]. The paraventricular nucleus of the mammalian hypothalamus (PVH) is the central localisation of melanocortin action. The melanocortin system components are crucial for energy homeostasis. In turn, arginine vasopressin encoded by AVP gene is expressed in the mammalian PVH, and is induced by anorectic melanocortin agonist melanotan II (MTII). Pei et al. [42] examined the effect of AVP on feed intake by using a specific knock-out (KO) mice in combination with a viral vector to manipulate PVH-AVP, and stated that PVH-AVP neurons are critical components for transducing the anorectic effect of melanocortin agonists. The increased *AVP* expression in the hypothalamus was observed only in 3-week-old HGR cockerels with increased feed intake. Thus, a doubtful role of AVP as a mediator during the transduction of anorectic MTII action at this developmental stage in broilers, or different signalling pipeline of MTII in this context. The OXT gene encodes oxytocin that is expressed in the PVH, which was confirmed in mammals. Numerous studies reported an anorexigenic effect of oxytocin in rats [44, 45]. Early studies suspected that OXT action during appetite suppression displays in homeostatic modulation of sodium balance [46]. In turn, the recent research suggests that oxytocin limits food intake rich in sucrose by suppressing the reward pathway [43]. The increased *OXT* expression in the HGR group that was characterised by increased feed intake suggests for a different function of OXT during nutritional regulation. Moreover, in older 6-week-old broilers, 2-fold increased *SIM1* expression was observed. SIM1 encodes transcription factor single-minded 1 that was found to interact with oxytocin [47]. The authors suggested that SIM1-expressing PVN neurons regulate feeding in response to melanocortin signalling but not energy expenditure. Therefore in the mammals, SIM1 is believed to be an anorexigenic molecule. In addition, Michaud et al. [48] indicated that SIM1 upstream regulation of *POU3F2* expression is related to differentiation of the specific neuroendocrine lineage of the PVH and secretion of OXT. The present study, as a first report, shows that increased *SIM1* expression was positively correlated with body mass and feed intake in broilers what suggest the orexigenic effect of this molecule in avians.

The present study shows that numerous orexigenic and anorexigenic molecules examined previously in the mammals seems to play a different role in the regulation of birds feeding behaviour. Nevertheless, the activity of neuromedin U (NMU) and neuropeptide W (NPW) in cockerels seems to be in line with previous studies in the mammals, because in the hypothalamus of 3LGR birds increased expression of both neurotransmitters was observed. The anorexigenic action of NMU was examined previously by Wren et al. [49], who found that NMU injected into the PVN or acute nucleus immediately decreased food intake in rats. Moreover, NMU released in the hypothalamus is stimulated by leptin, which is the major anorexigenic molecule. It has been confirmed that in pigs, neuropeptide W increases the feed intake in the bright phase and inhibits it in the dark period [50]. Its function was not fully elucidated, and the authors suggested that NPW regulates feeding behaviour in the hypothalamus by accompanying other feeding-regulating neurons. The present results suggest that NMU and NPW actions in feeding behaviour suppression in birds are dependent on age.

## Conclusion

In the present study, low growth rate chickens with lowered feed intake showed increased expression of genes that are known as orexigenic molecules in the mammals. Our observation indicates for a little distinct feeding behaviour regulation at hormone gene expression levels in avian than it was described by numerous authors in the mammals. Moreover, we presented several candidate genes associated with the growth rate in broiler chickens such as *POMC, NMU, NPW, PMCH, GAL,* and *FOS* that are differentially expressed in the hypothalamus in response to variable growth. The findings narrow the search area for genetic markers, which could be used in the selection procedure, and to promote population homogeneity that determines poultry producers’ profits. The present findings enrich our knowledge in terms of the regulation of nutrition processes in the broiler hypothalamus showing interesting avian hormonal controls of growth and feed intake.

## Methods

### Birds used in the experiment

All conducted chicken trial was approved by the Approving Experiment Committee of National Research Institute of Animal Production (Kraków, Poland), and with the national authority according to the Polish Act on the Protection of Animals Used for Scientific or Educational Purposes of 15 January 2015 (which implements Directive 2010/63/EU of the European Parliament and the Council of 22 September 2010 on the protection of animals used for scientific purposes). Moreover, all procedures were following the guidelines and regulations of the Local Krakow Ethics Committee for Experiments with Animals.

The study included 36 Ross 308 cockerels (300 in total) that were hatched in a commercial poultry hatchery. Eggs were obtained from the Ross 308 parent stock farm located in Wolbrom (Poland). Chicks were delivered to the experimental farm of the National Research Institute of Animal Production located in Aleksandrowice (Poland). Birds were weighed on day 1st and day 14th. Based on broiler mass after 2nd week, they were classified into lighter and heavier groups (*n* = 36) that were kept in separate pens (*n* = 9) on the deep litter with electronically-controlled environmental conditions (temperature, air humidity, lighting regime). During test fattening, traits were measured, such as body weight gain, feed intake and feed conversion ratio (FCR). The cockerels were standardly fed complete starter, grower and finisher diets in the period between days 1–21, 22–35 and 36–45, respectively. In the particular nutrition periods, the diet contained 22, 20.5 and 20.5% crude protein, respectively; and 2990, 3130 and 3130 kcal/kg metabolizable energy, respectively. Feed and water were available ad libitum. Ross 308 chickens show intensive growth on the day 21 from hatching, and in two following weeks, they double their BW and then GR gradually decreases. The present study confirms the differences in the hypothalamic activity at the transcriptome level in HGR and LGR Ross 308 cockerels at these two crucial developmental stages; after week 3 and 6. Eighteen birds were kept until day 22 from hatching (after 3rd week), with 9 in the low growth rate (3LGR) and 9 in the high growth rate (3HGR) groups, and they reached a mean BW of 1.2 kg. The next 18 birds were kept until day 45 (6th week) in the 6LGR and 6HGR groups, and they reached a mean BW of 3.5 kg. Before the slaughter, birds were subjected to a 6-h feed withdrawal but had constant access to water. The birds were euthanized by decapitation after electrical stunning (150 mA, frequency of 200 Hz for 4 s). Then, the whole chicken hypothalamus was collected and stored as was described by Piórkowska et al. [19]. Carcass and growth traits were measured following the method described by Połtowicz et al. [51].

The statistical analysis between high and low growth rate chickens was estimated separately for 3rd and 6th-week old chickens. The data was analysed by the Student’s t-test using Statistica 13.1 (StatSoft Inc.). A significance level of *P* ≤ 0.05 was considered statistically significant.

### cDNA library construction

**Fig. 4:**
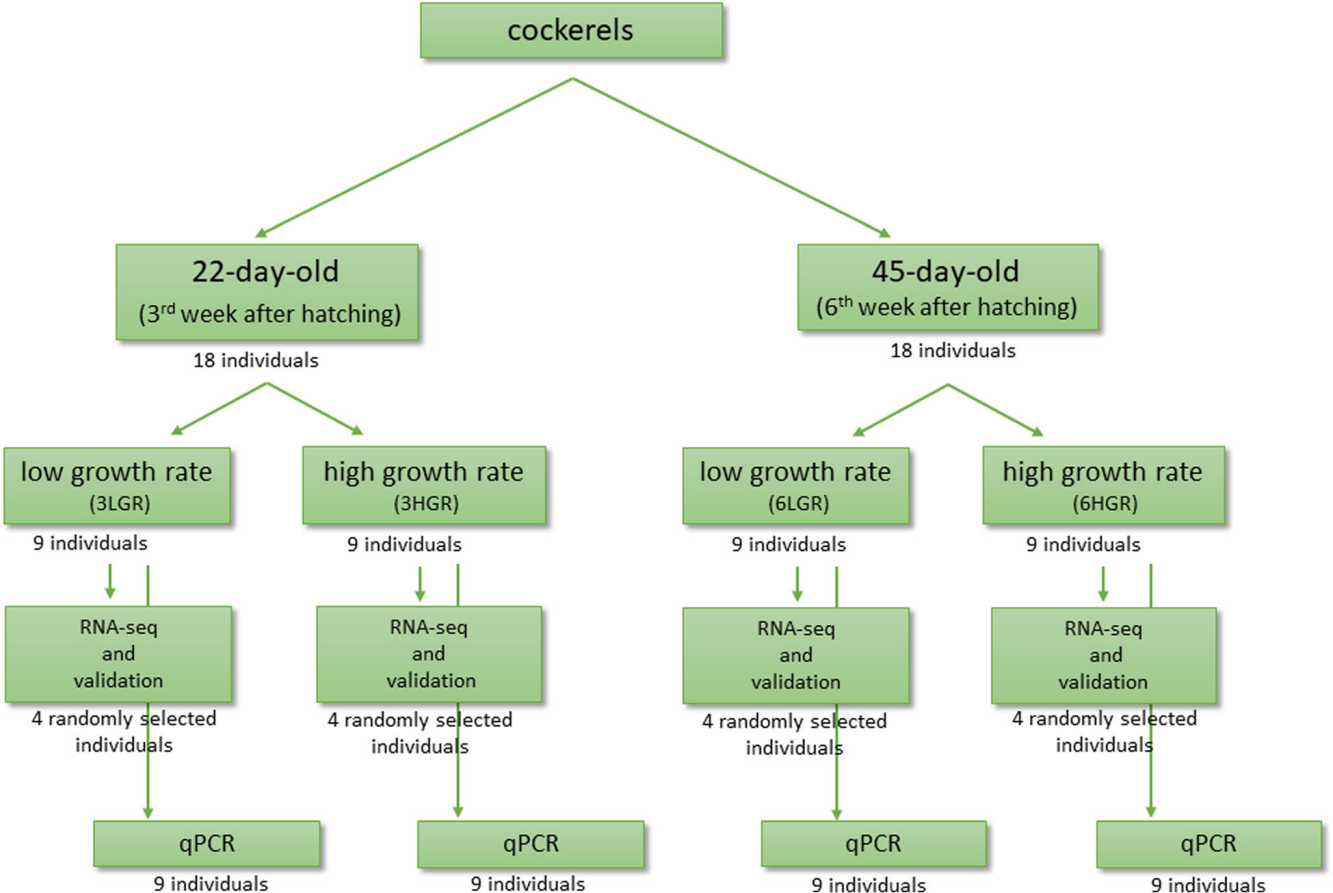
Bird selecting pathway for detecting the differentially expressed genes

RNA from hypothalamus was isolated, as was described by Piórkowska et al. [19]. The quality and concentration of RNA were measured using a TapeStation2200 (Agilent, Palo Alto, USA), and obtained RIN values were in the range between 7.3 and 8.4. RNA sequencing was performed for 16 randomly selected hypothalamus samples presenting HGR (*n* = 4) and LGR (*n* = 4) for both ages (Fig. 4). The cDNA libraries were prepared by TruSeq RNA Sample Preparation Kit v2 (Illumina, San Diego, CA, USA) according to the protocol and indexed with individual adaptors (File S1). The sequencing was performed on a HiScanSQ System (Illumina, San Diego, CA, USA) in 101 bp single end cycles using a TruSeq Kit v3-HS chemistry described by Piórkowska et al. [52].

### Alignment

Raw reads were processed using the FastQC, Flexbar and CASAVA version 1.8.2 programs (default parameters). The processed reads were aligned to the GCA_000002315.5 genome assembly (Ensembl Genome Browser). The alignment was done by RSEM, v. 1.3.0, and SAMStat software, and to generate statistics RNA-SeQC v1.1.8 (default settings) was used. The RNA sequencing data have been submitted to the Gene Expression Omnibus NCBI (accession number GSE104255).

### Differentially expressed gene analysis

Differentially expressed gene analysis was carried out using DESeq2 [53]. False Discovery Rate (FDR) was used as corrected *p*-values (≤ 0.05) due to multiple analysis. The threshold for gene expression fold change (FC) (comparison between HGR and LGR cockerels) was set at ≥1.5 level. The functional gene analysis was performed based on *G. gallus* reference using STRING v.10.5 and PANTHER™ Classification System v.14.0 tools (with default settings and established FDR at < 0.05 level).

### RNA-seq result validation

Eighth genes were validated by qPCR using *RPL4* and *SDHA* as endogenous controls [54]. qPCR reagents are presented in the supplementary material (File S2). Pearson’s correlation (SAS Enterprise v. 7.1) was used to compare RNA-seq and qPCR results. The genes for validation were chosen based on their relationship to the hormonal regulation.

The estimation of transcript abundance was performed for 36 cockerels at both ages (3- and 6-week-old chickens) and two GR groups (Fig. 4). The standard curve method was used to estimated qPCR. Relative transcript quantity (RQ) was calculated using delta-delta CT method [20], and significant results were determined by ANOVA (Duncan’s post hoc test; SAS Enterprise v. 7.1 with default settings; SAS Institute, Cary, USA).

The scheme of the whole experiment was shown in graphical abstract.

## Supplementary information

**Additional file 1: File S1.** Overall statistics and read annotations obtained for each library.

**Additional file 2: File S2.** Primers and probes used in the qPCR analysis.

## Data Availability

The data generated during the study have been deposited in the GEO (NCBI) under accession number GSE104255 available by link. https://www.ncbi.nlm.nih.gov/geo/query/acc.cgi?acc=GSE104255. Moreover, RNA-sequencing cleaned reads were aligned to the reference chicken genome GCA_000002315.5 available by link https://www.ensembl.org/Gallus_gallus/Info/Index. Functional analysis was perofroed using STRING: functional protein association networks v. 10.5 (https://string-db.org/) and PANTHER™ 14.0 (http://www.pantherdb.org/).

## Abbreviations

GR: Growth rate
NPY: Neuropeptide Y
ALDH1A1: Aldehyde dehydrogenase 1 family, member A1
GAL: Galanin
PMCH: Pro-Melanin concentrating hormone
GIEWS: Global Information and early warning system
DPM: Deep pectoralis myopathies
PSE: Pale, soft and exudative meat
SREBF1: Sterol regulatory element-binding transcription factor 1
LPL: Lipoprotein lipase
HGR: High growth rate
LGR: Low growth rate
DEGs: Differentially expressed genes
FCR: Feed conversion ratio
ACTH: Adrenocorticotropic hormones
αMSH: αmelanocyte-stimulating hormone
βMSH: βmelanocyte-stimulating hormone
ARC: Arcuate nucleus
BSX: Homeobox protein
POMC: Pro-opiomelanocortin
PVH: Mammalian hypothalamus
AVP: Arginine vasopressin
MTII: Melanocortin agonist melanotan II
OXT: Oxytocin
SIM1: Transcription factor single-minded 1
VP: Vasopressin
TRH: Thyrotropin-releasing hormone
CRH: Corticotropin-releasing hormone
GHIH: Somatostatin
NMU: Neuromedin U
NPW: Neuropeptide W
FDR: False discovery rate
