# Supplementary material for: Identification of candidate genes and regulatory factors related to growth rate through hypothalamus transcriptome analyses in broiler chickens
Source: BMC Genomics. 2020 Jul 23;21:509. doi: 10.1186/s12864-020-06884-5 (PMC7376931; doi:10.1186/s12864-020-06884-5)
Supplement: Supplementary file 1 — Additional file 1: File S1. Overall statistics and read annotations obtained for each library. [file 12864_2020_6884_MOESM1_ESM.docx]

| **Table S1.** Overall statistics and read annotations obtained for each library. | | | | | | | |  |
| --- | --- | --- | --- | --- | --- | --- | --- | --- |
| Sample | ROSS308_3LGR_1 | ROSS308_3LGR_2 | ROSS308_3LGR_3 | ROSS308_3LGR_4 | ROSS308_3HGR_1 | ROSS308_3HGR_2 | ROSS308_3HGR_3 | ROSS308_3HGR_4 |
| Indexes/ pooling lane1;3;5;7 | AR004 | AR005 | AR006 | AR007 | AR008 | AR011 | AR012 | AR013 |
| Total purity filtered reads sequenced | 22 368 454 | 23 487 271 | 21 195 496 | 21 760 511 | 24 100 292 | 23 697 330 | 25 871 916 | 25 222 717 |
| Alternative alignments | 1 235 584 | 1 439 409 | 1 307 849 | 1 390 977 | 1 481 718 | 1 428 338 | 1 559 170 | 1 510 397 |
| Exonic Rate | 0.70 | 0.69 | 0.68 | 0.70 | 0.69 | 0.69 | 0.69 | 0.69 |
| Intronic Rate | 0.090 | 0.088 | 0.086 | 0.078 | 0.080 | 0.082 | 0.085 | 0.087 |
| Transcripts detected | 28 206 | 28 317 | 28 017 | 27 898 | 27 968 | 28 153 | 28 215 | 28 060 |
| Genes detected | 10 338 | 10 349 | 10 289 | 10 283 | 10 299 | 10 309 | 10 342 | 10 296 |
| Base Mismatch Rate | 0.005 | 0.004 | 0.005 | 0.004 | 0.004 | 0.004 | 0.004 | 0.004 |
| **Alignment** |  |  |  |  |  |  |  |  |
| Number of input reads | 23 257 119 | 24 326 292 | 22 110 816 | 22 708 636 | 25027122 | 24532702 | 26819747 | 26169402 |
| % of uniquely mapped reads | 90.82 | 90.48 | 89.84 | 89.71 | 90.27 | 90.73 | 90.48 | 90.42 |
| % of reads mapped to multiple loci | 4.84 | 5.56 | 5.49 | 5.60 | 5.51 | 5.36 | 5.47 | 5.44 |
| Sample | ROSS308_6LGR_1 | ROSS308_6LGR_2 | ROSS308_6LGR_3 | ROSS308_6LGR_4 | ROSS308_6HGR_1 | ROSS308_6HGR_2 | ROSS308_6HGR_3 | ROSS308_6HGR_4 |
| Indexes/pooling lane1;3;5;7 | AR014 | AR015 | AR016 | AR018 | AR019 | AR020 | AR023 | AR027 |
| Total purity filtered reads sequenced | 23 481 476 | 21 886 178 | 23 142 988 | 21 459 556 | 23 441 751 | 20 409 838 | 22 747 714 | 24 845 405 |
| Alternative alignments | 1 329 734 | 1 265 177 | 1 362 205 | 1 276 773 | 1 389 046 | 1 189 051 | 1 336 307 | 1 419 671 |
| Exonic Rate | 0.70 | 0.70 | 0.70 | 0.69 | 0.69 | 0.69 | 0.69 | 0.69 |
| Intronic Rate | 0.084 | 0.084 | 0.086 | 0.086 | 0.084 | 0.084 | 0.086 | 0.086 |
| Transcripts detected | 28 288 | 27 982 | 28 226 | 27 911 | 28 130 | 27 727 | 27 975 | 28 046 |
| Genes detected | 10 325 | 10 264 | 10 332 | 10 236 | 10 307 | 10 223 | 10 283 | 10 298 |
| Base Mismatch Rate | 0.004 | 0.004 | 0.005 | 0.005 | 0.004 | 0.004 | 0.004 | 0.004 |
| **Alignment** |  |  |  |  |  |  |  |  |
| Number of input reads | 24 324 918 | 22 668 099 | 24 038 010 | 22 273 733 | 24329666 | 21233340 | 23586068 | 25760675 |
| % of uniquely mapped reads | 90.93 | 90.80 | 90.49 | 90.51 | 90.49 | 90.37 | 90.70 | 90.69 |
| % of reads mapped to multiple loci | 5.07 | 5.22 | 5.24 | 5.30 | 5.35 | 5.24 | 5.24 | 5.24 |
