# Supplementary material for: Identification of candidate genes and regulatory factors related to growth rate through hypothalamus transcriptome analyses in broiler chickens
Source: BMC Genomics. 2020 Jul 23;21:509. doi: 10.1186/s12864-020-06884-5 (PMC7376931; doi:10.1186/s12864-020-06884-5)
Supplement: Supplementary file 2 — Additional file 2: File S2. Primers and probes used in the qPCR analysis. [file 12864_2020_6884_MOESM2_ESM.docx]

| **Table S2.** Primers and probes used in the qPCR analysis | | | | | | | |
| --- | --- | --- | --- | --- | --- | --- | --- |
| Gene | | Primers and Probe  or  Taqman Gene Expression Assay ID | Amplicon length [bp] | Label | Reference sequences accesion numbers | PCR efficiency  % | Exon Boundary |
| *ALDH1A1* | Aldehyde dehydrogenase 1 family, member A1 | Gg03313288_m1 | 146 | VIC | NM_204577.4 | 100 | 6-7 |
| *ALDH6* | Aldehyde Dehydrogenase 1 Family Member A3 | Gg03362468_m1 | 146 | FAM | NM_204577.4 | 101 | 6-7 |
| *RPL4* | Ribosomal Protein L4  **housekeeping gene** | Gg03370187_m1 | 75 | VIC | NM_001007479.1 | 100 | 2-3 |
| *SDHA* | succinate dehydrogenase complex, subunit A,  **housekeeping gene** | Gg03330760_m1 | 82 | VIC | NM_001277398.1 | 100 | 5-6 |
| *CGA* | Thyroid-Stimulating Hormone Alpha Chain | F-AGGGAGAAAGATCATGGATTGC  R- GCAGAAATACAGACAAAATGGTCAA  probe - ACAGGAAGTATGCAGCTGT | 71 | NED | ENSSSCG00000025434 | 98 | 1-2 |
| *GHRH* | Growth hormone-releasing hormone | Gg03359589_m1 | 71 | FAM | NM_001040464.1 | 100 | 3-4 |
| *LECT2* | leukocyte cell-derived chemotaxin 2 2 | Gg03340174_m1 | 73 | FAM | NM_205478.1 | 100 | 5-6 |
| *POMC* | Proopiomelanocortin | Gg03352057_m1 | 134 | FAM | NM_001031098.1 | 100 | 1-2 |
| *OXT* | oxytocin | Gg03316427_m1 | 69 | FAM | ENSGALG00000019026 | 100 | 1-2 |
| *BSX* | Brain specific homeobox | Gg03365917_m1 | 63 | VIC | NM_204512.1 | 100 | 1-2 |
| *TBX3* | T-box 3 | Gg03330698_m1 | 66 | VIC | NM_001270878.1 | 100 | 1-2 |
| *PMCH* | Pro-melanin concentrating hormone | Gg03330394_m1 | 135 | FAM | NM_001195795.1 | 100 | 1-2 |
